# Editorial: Emerging engineering approaches in cancer immunotherapy

**DOI:** 10.3389/fonc.2022.1009604

**Published:** 2022-08-24

**Authors:** Jialu Xu, Xin Ming, Yuanzeng Min, Chao Wang

**Affiliations:** ^1^Jiangsu Key Laboratory for Carbon-Based Functional Materials and Devices, Institute of Functional Nano & Soft Materials (FUNSOM), Soochow University, Suzhou, China; ^2^Department of Cancer Biology and Department of Biomedical Engineering, Wake Forest University School of Medicine, Winston Salem, NC, United States; ^3^The First Affiliated Hospital of University of Science and Technology of China, Division of Life Sciences and Medicine, University of Science and Technology of China, Hefei, China; ^4^Department of Chemistry, University of Science and Technology of China, Hefei, China

**Keywords:** cancer immunotherapy, immunoengineering, Biomaterails, immune checkpoint block therapy, T cell therapeutics

As an unprecedented approach, cancer immunotherapy has transformed cancer treatment. However, only a minority of patients benefits from cancer immunotherapy. In order to improve the efficacy of cancer immunotherapy and reduce the occurrence of immune-related adverse reactions, emerging engineering approaches have been explored for cancer immunotherapy. This invited Research Topic is composed of 16 articles, including 4 original research papers, 8 review articles, 1 minireview article, 1 opinion article, 1 perspective article and 1 case report, contributed by a total of 109 researchers from all over the world (Total views: 53,987; as of July 26, 2022). This Research Topic covers a range of novel engineering approaches for cancer immunotherapy, including engineered T cells therapy (Xu et al.), bacteria-based synergistic therapy (Bao et al.), bioinspired membrane-coated nanoplatform (Mu et al.), and injectable hydrogel delivery system (Liu et al.), and so on.

Cancer has been threatening human beings with incurable, high mortality and high recurrence rate. Compared with non-tumor patients, tumor patients are more susceptible to SARA-Cov-2 and have poor prognosis (Huang et al.). Traditional therapeutic includes surgery, chemotherapy, radiotherapy, etc. To seek better treatment strategies, it is crucial to understand the mechanism of tumor occurrence and development. Overexpressed NPM1 promotes tumor growth. Liu et al. analyzed TCGA and GEO data and found that NPM1 is a prognostic biomarker related to immune infiltration in lung adenocarcinoma (LUAD), and is related to m6A modification and glycolysis. As an effective target for the diagnosis and treatment of LUAD, this provides a new strategy for the therapy of LUAD. Histone acetylation plays a role in regulating tumorigenicity, tumor progression, and tumor microenvironment. Xu et al. comprehensively analyzed 36 histone acetylation regulators in hepatocellular carcinoma (HCC) for the first time, and found a close correlation between histone acetylation patterns and tumor malignant pathways and tumor microenvironment, which is an important indicator for hepatocytes and provides new strategies for personalized and precise immunotherapy and prognosis of cancer.

So far, immunotherapy has a place in cancer treatment, such as the application of immune checkpoint inhibitors for HCC (Liu et al.). Combining traditional therapies with immunotherapy plays an important role in breast cancer (Zhang et al.). A case report has confirmed that combination of penpulimab and anlotinib can successfully treat extensive-stage small-cell lung cancer (ES-SCLC) (Zhang et al.). Engineered T-cell therapy includes adoptive T-cell therapy (ACT) (Xu et al.), among which chimeric antigen receptor T Cells (CAR-T) therapy has received extensive attention, especially in hematological tumors. Nonetheless, engineered T-cell therapy faces many challenges that hinder its clinical application. To accelerate the development of ACT, suitable experimental models and test platforms can be selected. Xiao et al. demonstrated that immunocompetent microphysiological system (iMPS) could triple-culture three-dimensional (3D) colorectal tumor microtissues, 3D cardiac microtissues, and human-derived natural killer cells in the same microfluidic network, and was able to simulate the *in vivo* state for corresponding tests. This provides new approaches for efficacy and early safety testing of new candidate for ACTs. For a more economically desirable effect, regenerable human induced pluripotent stem cells (iPSCs) were genetically engineered to differentiate into immune cells with enhanced antitumor cytotoxicity, increased persistence and decreased immunogenicity. CAR-T cells derived from iPSCs can be pre-prepared as off-the-shelf products and applied in a large number of patients, offering great promise for the next generation of ACT (Netsrithong et al.). CAR-T therapy can create new complications such as cytokine release syndrome, neurotoxicity, and even fatal cerebral edema. CD28-CAR heterodimerization may be an important cause of severe neurotoxicity (Ferreira et al.). To reduce its systemic toxicity, *in vivo* CAR-T cell therapy induced by gene editing tools can serve as a new generation of CAR-T cell therapy (Xin et al.). The development of CAR-T therapy in solid tumors is still in its infancy. By adopting some nanotechnology, such as nanozymes, RNA vaccines, etc., to help CAR-T cells target and accumulate in solid tumors, or to stimulate CAR-T cells by remodeling the tumor microenvironment, improve the survival rate and proliferation rate of CAR-T cells, and provide new ideas for the application of CAR-T cells in solid tumors (Mi et al.). Tissue resident memory CD8^+^ T (Trm) lymphocytes exist in various digestive tract cancers. CD8^+^ Trm cells own strong cytotoxicity, have ability to directly kill epithelial-derived tumor cells, and are important for maintaining the homeostasis of digestive tract mucosa and anti-tumor. But the application of CD8^+^ Trm cells in gastrointestinal cancers is still in its early stages. Specific drug therapy and cancer vaccine therapy targeting tumor-associated CD8^+^ Trm cells may become an important direction for precision cancer therapy (Mei et al.).

In addition to engineered T-cell therapy, other approaches have also been used to combat the challenges of cancer immunotherapy. Injectable hydrogel as a unique platform that can target the immunosuppressive tumor microenvironment have the advantages of good biocompatibility, good biodegradability and low toxicity (Liu et al.). Bioinspired membrane-coated nanoplatform have opened up novel research directions for cancer immunotherapy due to superior immune regulation and excellent tumor targeting (Mu et al.). The advantage of bacteria targeting tumor makes them an excellent platform for combination with immunotherapy. Optimizing bacteria-based therapy through strategies such as bioengineering or chemical modification can avoid the safety issues posed by this therapy (Bao et al.).

In general, this Research Topic reports the application of novel engineering approaches in cancer immunotherapy, which provides new ideas and strategies for cancer immunotherapy. Solving the challenges faced in cancer immunotherapy by various means has made an essential contribution to clinical translation and provides new hope for cancer patients.

## Author contributions

All authors listed have made a substantial, direct, and intellectual contribution to the work and approved it for publication.

## Acknowledgments

The editors appreciate the contributions of all authors to this Research Topic, the constructive comments of all the reviewers, and the editorial support from Frontiers throughout the publication process. This work was supported by Collaborative Innovation Center of Suzhou Nano Science & Technology, the 111 Project.

## Conflict of interest

The authors declare that the research was conducted in the absence of any commercial or financial relationships that could be construed as a potential conflict of interest.

## Publisher’s note

All claims expressed in this article are solely those of the authors and do not necessarily represent those of their affiliated organizations, or those of the publisher, the editors and the reviewers. Any product that may be evaluated in this article, or claim that may be made by its manufacturer, is not guaranteed or endorsed by the publisher.

